# T-Cell Artificial Focal Triggering Tools: Linking Surface Interactions with Cell Response

**DOI:** 10.1371/journal.pone.0004784

**Published:** 2009-03-10

**Authors:** Benoît Carpentier, Paolo Pierobon, Claire Hivroz, Nelly Henry

**Affiliations:** 1 Institut Curie, Laboratoire Physico-Chimie Curie, CNRS UMR 168, Université Paris VI, Paris, France; 2 Institut Curie, Laboratoire Immunité et Cancer, INSERM U 653, Pavillon Pasteur, Paris, France; New York University School of Medicine, United States of America

## Abstract

T-cell activation is a key event in the immune system, involving the interaction of several receptor ligand pairs in a complex intercellular contact that forms between T-cell and antigen-presenting cells. Molecular components implicated in contact formation have been identified, but the mechanism of activation and the link between molecular interactions and cell response remain poorly understood due to the complexity and dynamics exhibited by whole cell-cell conjugates. Here we demonstrate that simplified model colloids grafted so as to target appropriate cell receptors can be efficiently used to explore the relationship of receptor engagement to the T-cell response. Using immortalized Jurkat T cells, we monitored both binding and activation events, as seen by changes in the intracellular calcium concentration. Our experimental strategy used flow cytometry analysis to follow the short time scale cell response in populations of thousands of cells. We targeted both T-cell receptor CD3 (TCR/CD3) and leukocyte-function-associated antigen (LFA-1) alone or in combination. We showed that specific engagement of TCR/CD3 with a single particle induced a transient calcium signal, confirming previous results and validating our approach. By decreasing anti-CD3 particle density, we showed that contact nucleation was the most crucial and determining step in the cell-particle interaction under dynamic conditions, due to shear stress produced by hydrodynamic flow. Introduction of LFA-1 adhesion molecule ligands at the surface of the particle overcame this limitation and elucidated the low TCR/CD3 ligand density regime. Despite their simplicity, model colloids induced relevant biological responses which consistently echoed whole cell behavior. We thus concluded that this biophysical approach provides useful tools for investigating initial events in T-cell activation, and should enable the design of intelligent artificial systems for adoptive immunotherapy.

## Introduction

T-cell activation plays a central role in the mammalian immune response [1]. It is also the mainspring of several immunotherapeutic strategies [2], [3]. T cells are activated via engagement of T-cell receptors (TCRs) with antigenic peptides presented in the cleft of major histocompatibility complex (MHC) molecules at the surface of antigen-presenting cells (APCs) [4]. Activation occurs through formation of complex dynamic cell-cell contact, assembling several ligand-receptor pairs from key co-receptors to accessory molecules. Much progress has been made in recent years in describing the supramolecular organization of this cell-cell contact — the so-called “immune synapse” [5], [6], [7], and many facets of the signalling cascade are now clearly elucidated [8]. However, minimal requirements and relevant processes that link antigen recognition to downstream signalling remain unclear [9], [10], [11]. Gaining insight into the dynamic molecular complexity of whole cell-cell contact is a difficult challenge. We believe that a reductionist approach, using a simplified model presenting a cell on which the ligand nature and density are carefully controlled, could shed light on the relationship between molecular events and the cell response. Investigations using soluble ligands —although they have provided significant thermodynamic and kinetic data on molecular interactions at the cell surface — have clearly missed the 2D and collective nature of cell-cell contact. In order to take this into account, strategies consisting of replacing one of the cells in the interacting pair by a synthetic surface bearing appropriate T-cell ligands have been developed using either polymer microparticles [12], [13], [14], [15] or planar surfaces made up of supported lipid bilayers or monolayers on solid substrates [16], [17]. Although they constitute rather crude cell models, solid microspheres represent interesting investigative tools, since they enable exact specification of the nature and density of the ligand presented to the cell surface. To relate molecular bond formation at the cell surface to cell triggering, molecular binding and the cell response must be followed in parallel within the same time scale. One methodological approach, as used by Wei *et al.* [12], consists of using micromanipulation techniques to present the microsphere to the cell surface prior to imaging the cell response through intracellular cell calcium . This enables investigating the process at the single cell level and provides important qualitative information; however, it requires examining cells one by one, which is very time-consuming and limits the sample size, whereas variation between cells may be high. Thus, it cannot be easily implemented for examining several receptor classes or combinations, which is necessary for complex processes like T-cell activation.

Here we report a different approach enabling correlation of surface receptor engagement and the induced T-cell response through calcium rise monitoring on cell populations brought into contact, in suspension, with model grafted microspheres —the intracellular Ca^2+^ increase is taken as a reliable indicator of cell activation [18], [19]. We describe T-cell triggering by anti-CD3 grafted particles, confirming results previously obtained by others using imaging or functional methods to elucidate the ability of surface-immobilized anti-CD3 to activate T cells. Next, we explored induced signal properties and ligand density effects. We show that cell-particle contact stabilization is the limiting step in T-cell activation by these artificial systems in suspension. Using a ligand combination inspired by cell-cell conjugates, we coupled the LFA-1 adhesion molecule ligand to the microsphere surface and we demonstrated that this enables both overcoming and exploring contact limitations observed at low ligand density. Results consistently echo whole cell-cell behavior [20], [21], [22], supporting the validity of this approach for both dissecting the link between surface molecular interactions and T-cell triggering, and developing efficient artificial T-cell activation strategies.

## Results

### Anti-CD3-grafted particle binding to the T-cell surface

#### Binding profiles

In order to engage the TCR/CD3 receptor in well-defined and controlled conditions, we first prepared and characterized anti-CD3-coated micrometric particles. Then, to describe the level of cell receptor engagement, we examined T-cell-particle association properties — contact number and kinetics. Streptavidin-grafted particles were coated with biotinylated anti-CD3 monoclonal antibodies (mAb). Using the fluorescent titration procedure described in the methods section, we found an anti-CD3 surface density, ρ_max_, of (1.9±0.3)×10^4^ mAb/µm^2^, i.e.(4.8±0.5)×10^5^ /particle. This corresponded to a mAb to streptavidin average ratio equal to 1/3 in saturation conditions. This is consistent with the hypothesis of statistical spatial distribution limited by steric hindrance resulting from mAb size (MW≈180 000). Because a biotin-antibody chemical link was made up of a dozen sp^3^ carbons, we assumed that mAb molecules which bound to the particle via the biotin anchor were free to rotate so as to find their target on the cell surface. In contrast, as soon as one mAb binding site is engaged with its target on the cell surface, re-orientation should be hampered, very likely preventing engagement of the second binding site of the particle-grafted molecule. We then considered that on an average, only one cell receptor could be engaged by one mAb grafted on the particle surface.

Anti-CD3 particles coupled with anti-CD3 mAbs at saturation — ligand density, ρ = ρ_max_— were brought into contact with cells in HBSS buffer at concentrations equal to 2×10^7^/ml and 2×10^6^/ml, respectively. Contact was made under gentle stirring, producing random collisions between cells and particles under mild heterogeneous shear stress on the order of 1 to 10 dyne/cm^2^. This was estimated by tube diameter and stirring speed, giving fluid velocity induced by stirring (*V*≈1 cm/s), size of the cell particle conjugate (*h*≈10 µm) and fluid viscosity (water, η = 1cP). Shear stress is given by 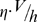.

**Figure 1:**
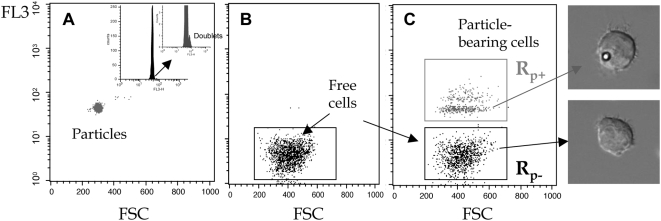
Cell-particle binding detection. Fluorescence in channel 3 (FL3, >670 nm emission) versus forward scattering dot plots of (A) particles alone, — corresponding histogram shown in insert , (B) cells alone, and (C) cells brought into contact with particles. Particle-bearing cells concentrated at higher fluorescence, gate R_p+_ are clearly distinct from free cells, gate R_p−_. Optical microscopy images illustrate each gate content.

**Figure 2:**
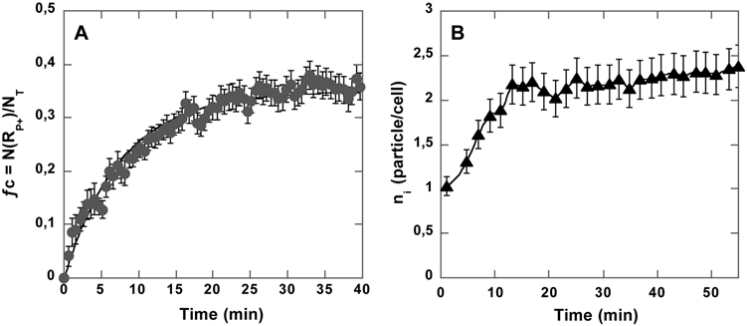
Cell-anti-CD3 particle binding kinetics. ƒ_c_, the ratio of the number of particle-bearing cells (number events in R_p+_) to the total number of cells N_T_ is shown as a function of time (A). 2×10^6^/ml T cells were brought into contact with 2×10^7^/ml anti-CD3 grafted particles under mild stirring. Experimental points (▴) were adjusted according to ƒ_c_(t) = N_max_/N_T_(1−exp(kt)) (solid line). Time dependence of the mean number of particles bound per cell (n_i_) is given in B.

Aliquots from this incubation tube were taken at regular time intervals and analyzed in flow cytometry (FCM). Due to particle residual fluorescence, cell-particle binding was clearly shown in FL3/FSC dot plots by emergence of a new cell population, gate R_p+_, at higher fluorescence (Fig. 1). From these data, we derived two parameters for describing the cell-particle association: ƒ_c_, the ratio of the number of particle-bearing cells (number events in R_p+_) to the total number of cells, N_T_, and *n_i_*, the mean number of particles bound per cell within the positive population obtained from FL_3_^+^, the mean FL3 value of the cells in gate R_p+_ and fl_3_ the fluorescence of one particle. Both kinetics are shown in Fig. 2. The cell-particle association levelled off for a fraction of cells having trapped particles, with ƒ_c_ close to 0.3 after 15 min incubation (Fig. 2A). The curve was adjusted to a first order monoexponential shape with a time constant *k* equal to 0.11±0.03 min^−1^, i.e. a half-time process of t_1/2_ = 6.3 min. The mean number of particles per cell reached a plateau in between 2 and 3 particles per cell with similar kinetics. Ungrafted particles brought into contact with cells under the same conditions did not display significant association with cells (maximum ƒ_c_ around 0.02). In order to check that cell-particle conjugates were not partially disrupted by shear stress undergone in the course of flow cytometer, we took several sample counts by microscopy. One-hundred cells were counted for each sample. We compared flow cytometry and microscopy counts both a short time after cell-particle contact — five min — and at the kinetic plateau. The percentage of particle-bearing cells was found equal to 11±2% and 34±3%, respectively, which confirmed results obtained by flow cytometry. These results indicated that specific binding actually occurred between anti-CD3 synthetic particles and Jurkat cells, but that only a fraction of cells was able to associate with a particle and that only a limited number of binding events occurred per cell.

#### Binding cut-off driving force

**Figure 3:**
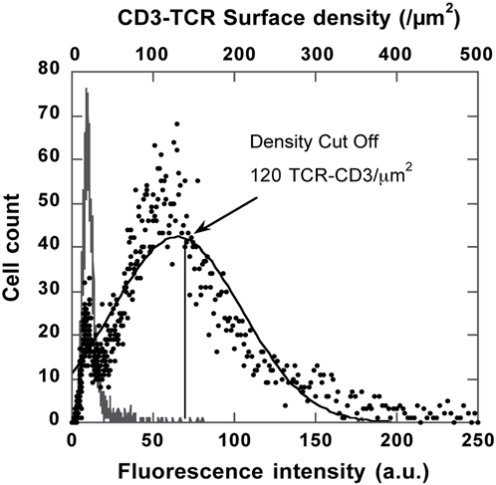
TCR/CD3 distribution. Cells (2×10^6^/ml) were labelled at 25°C or 37°C for 30 min using 5 nM FITC-anti-CD3 (•) or FITC-anti-CD19 as a negative control (—) in PBS buffer. Cell receptor distribution, reported by fluorescence intensity in channel 1 (FL1) shows a fraction of unlabelled cells of about 25%. Labelled cell distribution was adjusted to Gaussian distribution (
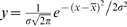
) (solid black line). Vertical line marks the limit for the 30% highest fluorescence right wing of the distribution.

In order to understand the origin of binding limitations observed, we first examined TCR/CD3 distribution over the cell population using FITC-anti-CD3 mAbs (Fig. 3). We systematically observed a 20% to 25% cell subpopulation devoid of labelling. Remaining cells were distributed according to a nearly Gaussian shape around a mean value of fluorescence FL1. Titration of this mean using a range of FITC-anti-CD3 concentrations indicated a mean number of TCR/CD3 per cell equal to 1.2±0.2×10^5^ per cell or 100 /µm^2^; the cell geometric surface was calculated using a mean radius of 7.4 µm and the effective surface area increased by a factor of 1.8 to account for membrane folds (42). When we performed titration on paraformaldehyde (PFA)-fixed cells in order to quench receptor internalization, we measured a higher number of receptors, which had increased by 1/3, indicating that partial internalization occurred upon anti-CD3 binding. Moreover, the mean number of particles per cell, *n_i_* increased significantly on fixed cells, suggesting that partial TCR/CD3 internalization, i.e. a mean cell surface density decrease, might control the number of binding events. In addition, comparison of TCR/CD3 distribution on a control cell sample (i.e. total cells) and on free cells of a cell-particle sample, showed that free cells consistently displayed lower mean surface density than total cells, indicating that the cell subpopulation which gained particles was a subpopulation with higher TCR/CD3 surface density (see Fig. S2). Taken together, these results argue for cell particle binding requiring minimal cell surface density of receptors. If we link TCR/CD3 distribution to the fraction of cells competent for particle binding, we found that a minimum number of receptors per cell equal to 1.4±0.2×105 was required in order for a cell-particle association event to occur. This corresponded to a surface density cut-off σ_exp_ = 120/µm^2^ (see Fig. 3). This may be understood in the theoretical framework developed by Cozens-Roberts et al., showing how receptor/ligand molecular bonds compete with mechanical forces in a hydrodynamic shear field to maintain a particle specifically bound to a surface through molecular links. In this case, competition may have originated when the suspension was stirred, which produced shear stress and tensile forces upon cell/particle contact. In the physical model, if we equate tensile forces due to shear stress with the strength of the molecular bonds, we note that a minimum number of bonds (N_th_) is necessary for stabilizing particle/surface contact [23]:
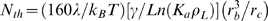
(1)where *λ* is the range of the interaction, *k_B_* is the Boltzman constant, *T*, the temperature, is shear stress, *K_a_* is the 2D association constant of the binding link, *ρ_L_* is the ligand surface density, *r_b_* is the radius of the particle and *r_c_* is the radius of the contact area. Applying this simple physical model to describe cell-particle contact formation over a short time and taking λ = 5×10^−8^ cm (given by Cozens-Roberts et al. [23] for an antigen-antibody bond), *K_a_* = (6±0.8)×10^18^ (mole/cm^2^)^−1^ (calculated from the 3D affinity constant determined experimentally for binding of UCHT1 anti-CD3 to the cell surface, (6±0.8)×10^9^ M^−1^, and a characteristic length equal to 10 nm to convert it to a 2D constant [24]), *ρ_L_* = 1.9×10^4^/µm^2^, *r_b_* = 1.4×10^−4^ cm, γ = 5 dyne/cm^2^, we calculated the minimum number of links required to stabilize the particle at the cell (from N_th_, we wrote a limiting surface density σ*_th_* = *N_th_/a* with *a* the contact area, related to *r_c_* by 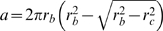 for a contact assumed to form a spherical cap. This surface density σ*_th_* was thus identified as σ*_exp_*, the experimentally determined density cut-off (σ*_exp_* = 120 molecules/µm^2^); we then numerically derived the value of *a* and calculated *N_th_*). This simple evaluation, assuming homogeneous molecular surface distribution, provided a minimum number of bonds on the order of 10, consistent with a 0.08 µm^2^ cell-particle contact area during the time of the collision. Although the Cozen-Roberts model was developed for ideal solid surfaces grafted with receptors and ligands, it convincingly describes the cell particle binding profile, at least qualitatively.

### Intracellular calcium wave stimulation

#### Particle-induced cell response

**Figure 4:**
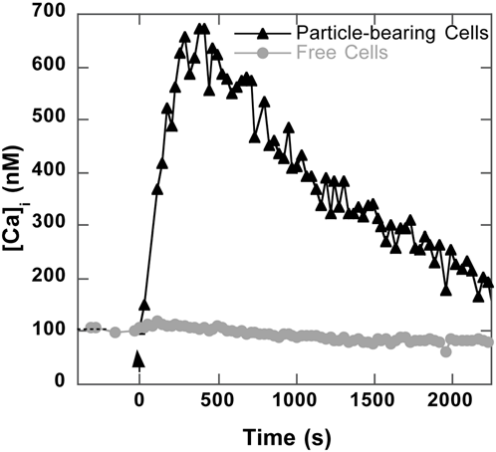
Calcium signal triggered by anti-CD3 particle binding. T cells loaded with the 0.5 µM Fluo-3 calcium probe were placed in contact with anti-CD3 grafted particles (*ρ_L_* = 1.9×10^4^/µm^2^). The intracellular calcium concentration [Ca^2+^_i_] was obtained from FL1 fluorescence intensity (see Materials and methods) for particle-bearing cells (▴) and free cells (•) identified by their respective FL3 intensity. This is a representative experiment out of more than four.

In order to evaluate the biological effect of this local 2D molecular contact, we investigated the cell response by concurrently monitoring the intracellular calcium concentration. Its rapid increase is one of the earliest markers of the biochemical cascade initiated in activated T cells [19]. Cells and anti-CD3-coated particles were brought into contact at 37°C, as above for binding experiments, except that cells had been previously loaded with the intracellular calcium probe Fluo-3. Flow cytometry recordings taken at regular intervals enabled collection of synchronized data reporting both particle binding (FL3 values) and the cell intracellular calcium concentration (FL1 values). FL3 values reported cell-particle association and enabled discriminating between particle-free cells and particle-bearing cells, and FL1 values provided related Ca^2+^ intracellular concentrations according to the calibration procedure described in Materials and methods. Cells and particles were bound as described above, and we observed that cells forming stable contact with particles displayed a fast-rising transient Ca^2+^_i_ signal (Fig. 4). Cells devoid of particles did not show any Ca^2+^_i_ changes, indicating that only stable contact, but not transitory collision, was able to trigger an intracellular calcium increase. Detailed analysis of particle-bearing cell population FL1 versus FL3 fluorescence enabled identifying single-particle-bearing cells (see Fig. 1), clearly showing that only one contact was needed to induce the transient calcium rise. Ungrafted particles did not induce intracellular calcium modifications, even in the few background cells that had non-specifically trapped a particle.

#### Soluble anti-CD3-induced calcium signal

**Figure 5:**
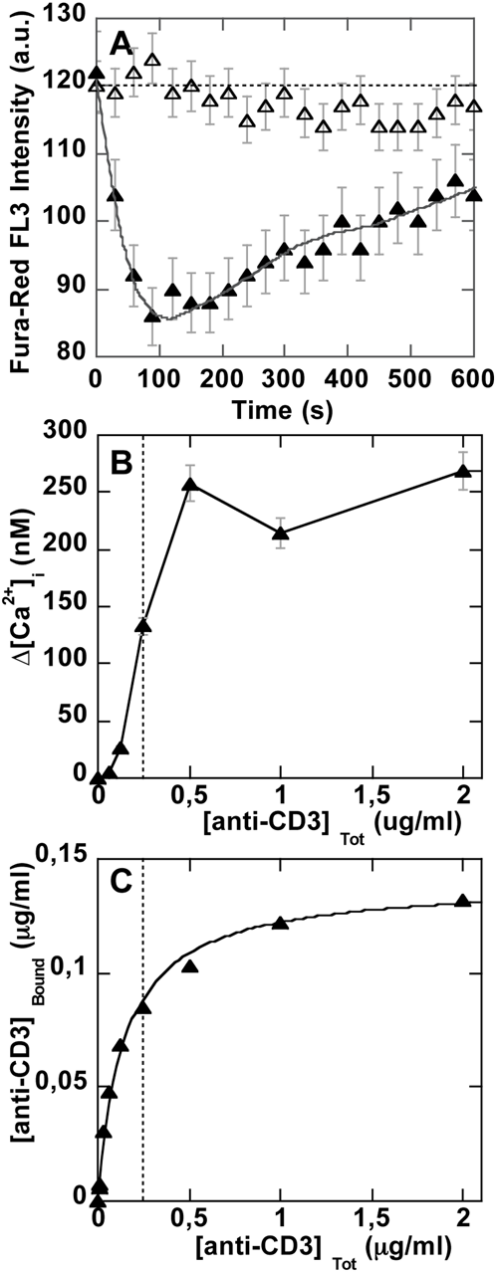
Cell TCR/CD3 occupation and calcium signal triggering by soluble anti-CD3. T cells (2×10^6^/ml in PBS buffer) loaded with 10 µM Fura-Red calcium probe were treated with increasing concentrations of soluble FITC-anti-CD3 (0.075 to 2 µg/ml) at time t = 0. (A) Time-dependent Fura-Red fluorescence intensity variation is shown for 2 µg/ml (▴) and 0.075 µg/ml (Δ) anti-CD3. (B) The corresponding intracellular calcium changes are reported as a function of anti-CD3 concentration. (C) Anti-CD3-FITC binding curve, obtained simultaneously with mean FL1 fluorescence intensity.

To compare the properties of a signal induced by focal binding of grafted particles with the signal induced by soluble anti-CD3 under the same conditions, we performed an experiment enabling monitoring of both anti-CD3 binding, using FITC-anti-CD3 emitting in an FL1 channel, and Ca^2+^_i_ changes using a Fura-Red calcium probe emitting in the FL3 channel. Results shown in Fig. 5 demonstrate that a calcium transient rise was triggered by soluble antibodies (Fig. 5A) in an all-or-nothing process (Fig. 5B) above an anti-CD3 concentration threshold equal to 0.125 µg/ml. As seen in Fig. 5C, this concentration corresponded to cell TCR-CD3 receptor engagement close to saturation. The calcium rise had a peak intensity around 120 s for an intracellular calcium concentration approaching 300 nM (calculated from Fura-Red intensity and calibration). The same experiment performed with a Fluo-3 calcium probe and non-labeled UCHT-1 antibody provided the same Ca^2+^_i_ signal characteristics.

#### Comparison of colloidal versus soluble anti-CD3-induced calcium signal

**Figure 6:**
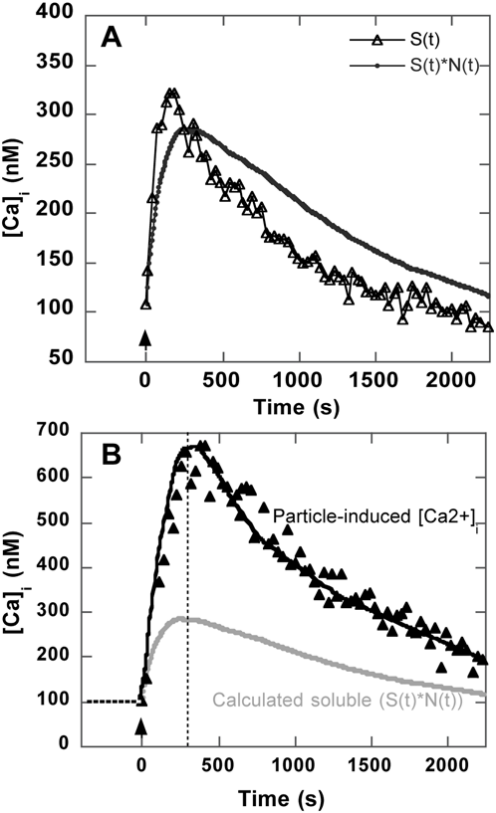
Comparison of colloidal versus soluble anti-CD3-induced calcium signal. (A) *S(t)* (Δ), the calcium signal induced by 0.5 µg/ml soluble anti-CD3, is displayed with *C(t)* (•), the convolution of *S(t)* with the time function reporting growth of the particle-bearing cell population, *N(t) = N_max_(1−exp(−kt)* using *k* = 0.11 min^−1^. (B) The convoluted signal C(t) (•) is shown with calcium signal induced by particles. Maximum signal is obtained near 300 s (indicated by dashed line).

At first sight, a soluble anti-CD3-induced Ca^2+^_i_ signal appeared to display a lower rise in amplitude and faster kinetics than the signal obtained using focal engagement of TCR/CD3 by a particle. Yet, in order to be able to compare the two signals, it was necessary to take into account the discrete time-dependent engagement of TCR/CD3 receptors by particles within the population of particle-bearing cells. This process was reported by the time function N(t) = N_max_(1−exp(−*k_t_*t)) (see Fig 2 and text above). In contrast, soluble anti-CD3 was received by all cells at the same time. In standard FCM analysis, the signal is averaged over the whole R_p_^+^ population independently of differing cell signal desynchronization. Due to noticeable signal noise, colloid-induced signal deconvolution using binding kinetics appeared to be inaccurate. We thus decided to proceed the other way around by convoluting the calcium signal elicited by soluble ligands, S(t), with binding kinetics, and N(t), generating a signal C(t) directly comparable to a particle-induced signal (Fig. 6). The combination of S(t) with binding kinetics induced both a slowdown and an amplitude decrease in the signal (Fig. 6A), depending on the respective time constants of S(t) and N(t) (see supplementary data, Fig. S1). Eventually, this signal displayed a twofold lower amplitude than the particle-induced Ca^2+^_i_ signal, but very similar kinetics, with the peak signal occurring, in both cases, at around 250±10 s (Fig. 6B).

These results confirmed that focal engagement of cell receptors by surface-bound ligands has greater efficacy than do soluble activators, but results also show that calcium rise kinetics were similar for both modes of activation, suggesting that the same signaling cascade was engaged.

### Interaction and signal control

#### Decreasing particle ligand surface density

**Figure 7:**
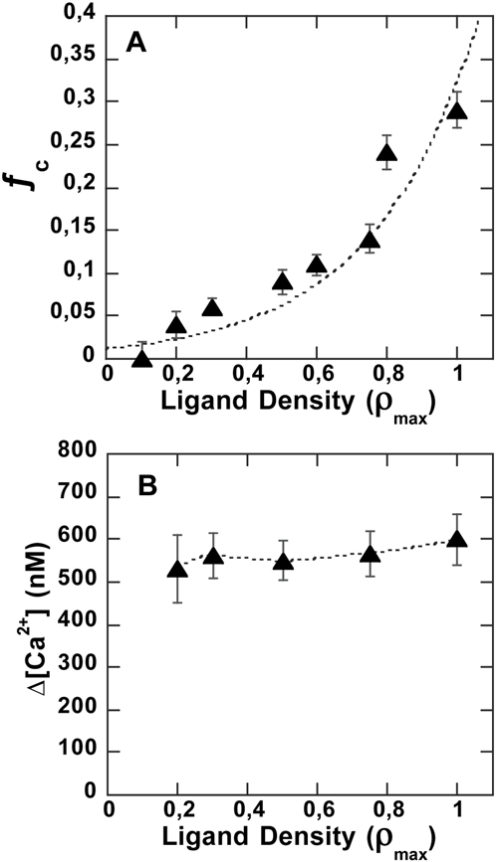
Anti-CD3 particle surface density variation. (A) Fraction of particle-bearing cells as a function of anti-CD3 ligand density on the particle surface. (B) Maximum calcium concentration induced by particle contact as a function of particle grafting density. Conditions as in fig. 2.

Next we sought to determine how calcium triggering depended on the number of engaged cell receptors, by changing ligand density on the particle surface ρ_L_. We prepared a series of particles grafted with decreasing concentrations of ligand and brought them into contact with cells in order to monitor induced intracellular calcium changes. The results displayed in Fig. 7 present both the binding fraction and the induced calcium rise obtained as a function of ρ_L_. The reduction in ρ_L_ caused a quasi-exponential decrease in binding efficiency, as reported by ƒ_c_ — no cell-particle binding was observed for grafting densities below 1/5 saturation density. However, as long as binding events were obtained, the triggered calcium signal displayed optimal amplitude and kinetics at all ρ_L_ values. This was consistent with the idea that a minimum number of bonds (about 10) must form in order to create cell-particle contact, implying, reciprocally, that all formed contacts have gathered this minimal number of links and are thus logically able to support a full calcium rise, although with a lesser number of cells as ρ_L_ decreases. To further explore the relationship between the number of engaged receptors and signalling efficiency through calcium rise, we decoupled TCR/CD3 engagement and cell-particle contact formation. For this purpose, we implemented a strategy inspired by the T cell itself using adhesion molecule LFA-1 to anchor the particle.

#### LFA-1 engagement for holding the particle at the cell surface

**Figure 8:**
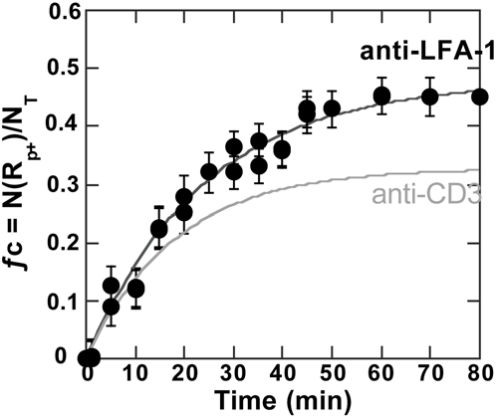
Cell-anti-LFA-1 particle binding kinetics. ƒ_c_, the ratio of the number of particle-bearing cells (number of events in R_p+_) to total number of cells N_T_ is displayed as a function of time. Conditions as in fig. 2 except that particles were grafted here at saturation with anti-LFA-1 (•) (ρ_L_ = 1.9×10^4^/µm^2^). The curve obtained with anti-CD3 particles was repeated to allow comparison (––).

We first grafted particles to saturation with an anti-LFA-1 mAb (CD18). Grafting was very similar to anti-CD3, providing a ligand number per particle close to (1.5±0.5)×10**^5^** anti-LFA-1 per particle. These particles were brought into contact with cells according to the same protocol as anti-CD3 particles, and association kinetics were monitored by flow cytometry as above. The binding profile showed an ƒ_c_ plateau value equal to 0.47 (Fig. 8). This was higher than the cell binding ratio obtained with anti-CD3 particles previously found around 0.3. In parallel, LFA-1 surface expression was stronger than TCR/CD3, *i.e.* (1.8±0.5)×10^5^ per cell or 160/µm^2^ versus 120/µm^2^, but the affinity of anti-LFA-1 grafted onto particles for its receptor was lower than that of anti-CD3 (K_a_(LFA-1) = 6×10^8^ M^−1^ versus K_a_(CD3) = 2×10^9^ M^−1^). Finally, this provided binding efficiency very close to those of anti-CD3 particles.

**Figure 9:**
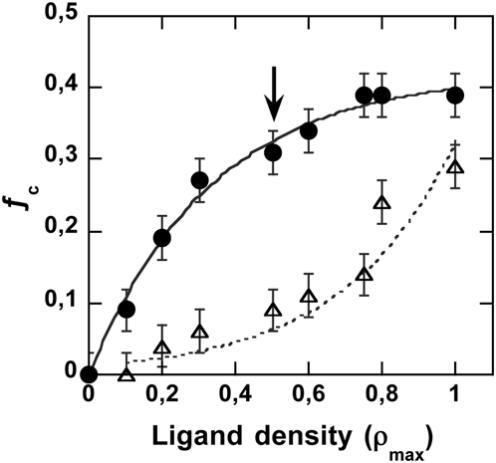
Anti-LFA-1 particle surface density variation. Fraction of particle-bearing cells as a function of anti-LFA-1 ligand density on the particle surface (•). Curve obtained with anti-CD3 particles at various surface densities was repeated for comparison (Δ). Conditions as in fig. 2.

Next we varied the anti-LFA-1 surface density of the particles and observed that the decrease in ρ_L_ clearly had a less drastic effect on cell recruitment than with anti-CD3 particles, possibly due to better exposure of the receptor at the cell surface, in good agreement with respective molecule morphology. LFA-1 displayed higher extension length above the cell surface than TCR/CD3 as shown by the size of the complexes they form with MHC and ICAM-1 — 40 nm compared to 15 nm [25]. A measurable binding ratio was still obtained for a surface density equal to ρ_max_/10 (Fig. 9). It was then possible to prepare hybrid particles bearing a stabilizing density of anti-LFA-1 and saving available functions to bind a large range of anti-CD3 densities. When these anti-LFA-1-grafted particles were tested for Ca^2+^_i_ stimulation, no Ca^2+^_i_ changes were observed, except for a slight increase in rare cases.

#### Anti-CD3 / anti-LFA-1 hybrid particles

**Figure 10:**
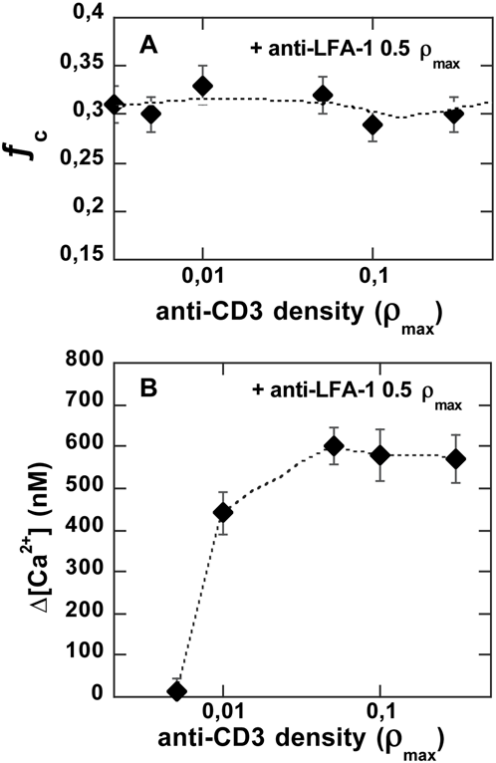
Calcium triggering using hybrid anti-LFA-1 and anti-CD3 grafted particles. (A) Cell-particle binding ratio, ƒ_c_, and (B) intracellular calcium changes induced by particles grafted with 0.5ρ_max_ anti-LFA-1 and anti-CD3 variable densities from 0.005 to 0.3 ρ_max_. Conditions as in fig. 2.

Particles were then grafted with an anti-LFA-1 surface density equal to 0.5ρ_max_ and various anti-CD3 densities ranging from 0 to 0.3ρ_max_. Following this, we tested their capacity to associate with T cells. A mean ratio of particle-associated cells equal to 0.3±0.05 was obtained for all samples independently of anti-CD3 density (Fig. 10A), indicating that hybrid-particle binding was dominated by engagement of LFA-1, with neither a positive nor a negative contribution of anti-CD3, at least to cell-particle conjugate formation.

We then monitored Ca^2+^ *_i_* in cells brought into contact with these hybrid particles. The results presented in Fig. 10B show that the calcium wave triggered by cell particle binding was maximal as long as particle anti-CD3 density was at least equal to 0.05 ρ_max_. The calcium rise profile was conserved; in particular, no sustained calcium increase occurred in the presence of anti-LFA-1 on the particle. At lower density, the mean calcium rise amplitude decreased due to the emergence of non-responding particle-associated cells, whereas responding cells exhibited the same calcium wave amplitude as cells bearing particles of highest anti-CD3 surface density.

We then grafted particles with 0.1ρ_max_ anti-CD3 density and varied the amount of anti-LFA-1 from 0.2ρ_max_ to 0.9ρ_max_. The calcium wave appeared to be drastically reduced for anti-LFA-1 densities above 0.7ρ_max_ (data not shown). This suggests that engagement of TCR/CD3 was in this case hampered by formation of a large number of adhesive links. This might also be due to triggering during strong LFA-1 engagement of a countersignal hampering Ca^2+^ increase pathway.

In order to gain further insight into this question, we treated cells with saturating concentrations of soluble anti-LFA-1 before bringing them into contact with anti-CD3-coated particles. Fraction of bound cells was significantly lower than in the absence of LFA-1, and no calcium signal from particle-bearing cells was detected. Results strongly support the hypothesis of anti-CD3-CD3 binding inhibition due to steric hindrance produced by a high density of LFA-1-anti-LFA-1 bonds. To test whether binding inhibition arose from CD3 exclusion from the contact zone, we made fluorescence images using ζCD3-GFP expressing Jurkat cells [26]. Fluorescent TCR/CD3 was then monitored as particle coated with anti-CD3/anti-LFA-1 (10/90 ratio) was brought into contact with these cells. The images shown in supplementary data (Fig. S3) did not evidence any CD3 exclusion in cell-particle contact. Steric inhibition of CD3 engagement by a high density of anti-LFA1 thus clearly seemed to be responsible for the observed cell response inhibition.

## Discussion

We describe here an experimental approach to exploring the link between cell surface molecular events and a short time-scale cell response statistically based on a large number of events. We implemented a strategy for investigating T-cell activation — a process involving close to ten ligand-receptor pairs. Defining the minimal requirements and discriminating between key and accessory events necessitates elucidation of the biological outcome of such receptor engagement independently or in combination using simplified artificial models.

We demonstrate here that model colloids grafted to target cell receptors may be efficiently used for this purpose. Solid particle systems combine the advantage of a 2D configuration with controlled surfaces having well-defined molecular composition and cell receptor focal engagement, together with the possibility of easily contacting a large number of cells at a time, bringing cells and particles into contact in suspension [27].

Several authors have used synthetic model systems, among them ligand-grafted particles for mimicking antigen presentation. Wülfing et al. [28] attached grafted beads to the lymphocyte surface to follow accumulation of molecules in T cell/APC contact upon T-cell triggering; Wei *et al.* [12] used optically trapped particles to map T-cell sensitivity in polarized lymphocytes. They also showed that T-cell activation could be obtained using a low level of TCR engagement, as also demonstrated in experiments on cell-cell conjugates [20], [21], [22]. These experiments were performed using single cell imaging and individual particle handling, providing valuable information at the single cell level, but intrinsically of low statistical weight and limited to small-sized samples.

Our experimental strategy consisted in following both binding and activation using flow cytometry analysis on populations of thousands of cells.

We first targeted TCR/CD3, which ensures MHC-antigenic peptide recognition. Receptor engagement was assessed by a stable cell-particle association and activation was evaluated on the basis of the intracellular calcium rise, known to induce distinct signaling pathways inside the cell, but which undoubtedly marks cell commitment to the activation process [19]. As expected, we observed that a specific cell-particle association induced a transient calcium signal, confirming previous results obtained by video imaging [12], [28] and validating our approach. The amplitude of the signal (maximal [Ca^2+^_i_ ]) was about twice as high as that produced by the same anti-CD3 in a soluble form consistent with previous observations on the higher efficiency of surface-bound [29] or cross-linked [30] ligands. The stable association of a single particle with the cell was sufficient to produce optimal Ca^2+^_i_ signalling. Our results established that the calcium rise induced by focal engagement of TCR/CD3 by colloids displayed the same kinetics as that induced by soluble ligands. This suggests that the same signalling pathway was elicited in both cases. Several years ago, Hashemi et al. [14] reported a more sustained Ca^2+^_i_ response using anti-TCR-coated beads instead of soluble ligands. Yet, although they targeted a different molecular subunit within the TCR/CD3 complex and used a different cell line, we suspect that the signal widening they observed with the coated beads might have been due to a de-synchronized Ca^2+^_i_ signal caused by cell-particle discrete and sequential binding.

Next, we decreased anti-CD3 surface density, inducing clear-cut decreasing T-cell/particle interactions. We suggest that binding limitation originates via competition between mechanical forces due to hydrodynamic shear stress and molecular bond strength. Using the model introduced by Cozens-Robert et al. [23], we found that, under our conditions, about ten bonds were required to stabilize cell-particle contact. This number depended on the shear stress level itself related to physical issues of the experimental configuration — stirring speed or vessel geometry. On the other hand, shear stress will also determine cell-particle collision duration, i.e. the time lapse during which the required bond series must form to stabilize cell-particle contact. This would be on the order of 5 ms for hard sphere collisions in a 5 dyne/cm^2^ shear stress according to Goldsmith and Mason [31]. In the case of cell-particle contact, collision duration might be longer due to cell elastic properties, surface roughness and transitory formation of a few bonds further dissociated by flow tensile strength. A compressive force regime lasting about ten milliseconds was used by Shankaran and Neelamegham in their work on neutrophil homotypic aggregation for shear stress in the range of 3 to 5 dynes/cm^2^. Considering a typical kinetic constant value of 10^7^ M^−1^.s^−1^ for the receptor-ligand association [32], we also calculated bond formation half-time in the range of tens of milliseconds, i.e. in the same range as the collision time. This indicates that the collision time could be kinetically limiting for receptor-ligand bond formation, with the time that the receptor and ligand spend at binding distance being too short to enable bond formation.

The introduction of LFA-1 ligands on the particle surface overcame limitations brought about by TCR/CD3 contact stabilization requirements and helped to elucidate low TCR/CD3 surface engagement densities which did not support cell-particle contact stabilization. Grafted alone, anti-LFA-1 ligands produced binding profiles similar to those of anti-CD3, except that a higher cell fraction was recruited for the same particle surface ligand density.

Using LFA-1 to anchor the particle onto the cell surface, it was possible to strongly decrease anti-CD3 density without affecting the calcium response. For instance, a 0.01ρ_max_ surface density in anti-CD3 still triggered an optimal Ca^2+^_i_ signal. Yet, in this extreme case, only two-thirds of the particle-bearing cells displayed an optimal calcium rise, indicating that the limit of triggering density was reached. In our system, ligands are tightly bound to the particle surface and the grafting density sets the intermolecular distance (close to 70 nm in the case of 0.01 ρ_max_). We suggest that the measured limiting density might correspond to minimal co-localization conditions needed to trigger T-cell activation through TCR engagement, describing a maximal distance so as to initiate the intracellular signalling cluster. The combined targeting of LFA-1 and TCR/CD3 consistently echoed the biological situation in which LFA-1/ICAM-1 increasingly appeared to impact T-cell activation through increasing contact duration, as, for instance, in the recent report by Scholer et al. [33].

Due to the tight binding of T-cell ligands to the particle surface, T-cell activation triggered here occurred in the absence of clustering and lateral compartmentalization of engaged molecular bonds. Although this type of spatial rearrangement has been extensively described following TCR engagement in cell conjugates and model systems, it is not quite clear whether differential clustering itself impacts the functional response of T cells, prolonging or contributing to extinguishing signalling [34], [35], [36], [37], [38]. In the system shown here, ligand immobilization does not prevent cell triggering. However, it remains possible that on the cell side, receptors keep on diffusing, alternatively shifting from a bound to a free state according to their k_on_, k_off_ and diffusion coefficients.

Thus far, we should mention that even in our simplified experimental model, several unresolved questions remain. Although we were able to determine a lower limit of surface density, additional investigations are needed to determine the exact number of bonds actually formed when such molecular densities are brought into contact. To this aim, both simulations and experiments on model 2D molecular networks describing the statistics of bond formation as a function of surface densities would be helpful. A better knowledge of the mechanism of contact formation and spreading would be useful as well. Indeed, although using the model of Cozens-Roberts et al. [23], we derived a nucleating contact area of 0.08 µm^2^, it could be observed on microscope images that this initiating contact quickly (in less than 50 ms) spread to a larger contact area — between 0.5 and 2 µm^2^ (see supplementary data, Fig. S4). Control of the contact area through, for instance, high throughput micro-fluid devices controlling both cell-particle time contact and contact area, could be an interesting trail to explore.

In addition, T-cell activation is also scrutinized for immunotherapeutic applications such as adoptive cell transfer, which achieves T-cell stimulation and expansion *ex vivo* before transferring them back to the patient. This requires efficient methods for generating large numbers of competent T cells. Cell-based strategies involving engineered antigen-presenting cells [39], [40], [41] have provided promising results demonstrating cancer regression mediation [2], [42]. However, extension of such strategies under reproducible clinical conditions at acceptable cost and time lapse remains a major challenge, and development of a-cellular systems offers an attractive alternative [43]. Currently, the efficiency of these systems is evaluated by adding artificial antigen-presenting systems to T cells and counting the number of competent cells produced after several days of co-culture. This is of utmost importance for the crucial step of patient re-infusion, but represents a long and cumbersome process not well-adapted to screening receptors and receptor combinations, a necessary step for optimizing artificial activation system coating. The approach shown here, enabling rapid association of particle coating with T-cell triggering efficiency in parallel with cell binding efficiency, would help in the developmental phase of new synthetic systems, by addressing unresolved questions such as that of the ideal combination of receptors to be engaged by the artificial system [41], [44], [45], [46].

### Conclusion

In this work, we have detailed the bases of collective engagement of T-cell surface receptors using synthetic colloids with the appropriate molecular surface engineering. Cells and particles were brought into contact in a dynamic configuration to allow analysis of populations of thousands of cells. Despite the simplicity of their conception, they induce relevant cell responses and appear to be valuable tools for exploring the links between cell surface receptor engagement characteristics and cell responses, especially in the case of T-cell activation where several receptors need to be evaluated separately and in combination.

Under dynamic conditions, inherent hydrodynamic shear stress determines contact nucleation requirements, which represent the limiting step in overall activation. Introducing adhesion molecules at the particle surface, just as nature does, enabled overcoming this. The present work highlights the importance of the cell-particle contact mode in the overall efficiency of an artificial T-cell activation process. We argue for taking this into account in the design of intelligent artificial antigen-presenting cells for adoptive immunotherapy. For this purpose, we suggest that use of high-throughput microfluid technology for monitoring physical parameters of cell-particle collision, such as contact time and contact area, will be highly valuable for further developing these artificial activation systems.

## Materials and Methods

### Cells

Wild-type Jurkat cells (clone 20; obtained from Dr. A. Alcover, Pasteur Institute, Paris, France) were grown in glutamax-containing RPMI 1640 (Invitrogen Life Technologies, Carlsbad, CA) supplemented with 100 U/ml penicillin, 100 µg/ml streptomycin, and 10% FCS (fetal bovine serum E.U approved Origin, Gibco Invitrogen). HBSS and PBS used for cell labeling were from Invitrogen.

### Reagents, buffers and antibodies

Fluo-3, Fura-Red, A-23187 and the protein-biotin coupling kit (Molecular probes F-6347) were purchased from Invitrogen. The intracellular calcium calibration kit which contains prediluted buffers of defined free Ca^2+^ concentrations ranging from 0 to 39 µM fixed by adequate concentrations of EGTA was from Molecular Probes. The following mAbs were used: purified or labelled with fluorescein isothyocyanate (FITC) or phycoerythrin (PE)) anti-human CD3 (clone UCHT1) and anti-human LFA-1 (CD18, clone 6.7 targeting integrin β2 chain) were from BD Biosciences Pharmingen (Le Pont de Claix, France). F(ab)^'^_2_ goat anti-mouse fragment IgG (H+L) (GAM) labelled with Alexa Fluor 488 was from Invitrogen.

### Particles and coatings

Streptavidin particles of 2.8 µm diameter were purchased from Dynal (Compiègne, France). Particles — typically 200 µl, 5×10^7^/ml — were coated for 30 min at 25°C in PBS buffer with 2 µl of 0.33 mg/ml anti-CD3 or anti-CD18, previously biotinylated using the Fluo Reporter Mini Biotin XX protein labeling kit and then washed twice in PBS. Alternatively, antibody was directly grafted on carboxylated particles using carbodiimide according to a simple procedure already detailed in Lebœuf and Henry [47]. No significant difference was observed between particles prepared by either procedure in the amount of associated ligands or stability of the coating. Particle final concentrations were adjusted using Malassez counting.

### Flow cytometry

Flow cytometry data were acquired using a Becton-Dickinson FACScalibur equipped with an air-cooled 488-nm argon ion laser. Fluorescence was collected using dichroic mirrors and filters sets: a 530/30 nm band pass on FL1 channel, 650 nm long pass on FL3 channel. In general, 5000 events were collected. Data were analyzed using multivariate analysis CellQuest (BDIS) and FlowJo software.

### Titrations

Fluorescence absolute calibration was performed using an autocalibration method detailed elsewhere [47], enabling linking mean fluorescence provided by the cytometer photomultiplier and the numbers of fluorescent-bound molecules per cell or particle. Briefly, proportionality was obtained directly from the slope of the titration curve, giving fluorescence per cell as a function of increasing fluorescent ligand concentration. In the initial linear part, the ligand concentration was low and receptors were in excess; for high affinities, the amount of free ligand may be neglected (less than 1% approximation, since the receptor concentration is higher than 100/K_a_). The amount of complex was thus very close to the total amount of ligand. This is consistent with our experimental conditions and avoided all drawbacks related to calibration performed with beads having optical properties different from those of cells. Using this principle, both cells and particles were titrated for their surface densities in receptors and ligands: TCR/CD3 and LFA-1 cell surface densities were obtained using FITC-anti-CD3 (UCHT1) and PE-anti-LFA-1, respectively. Titration curves giving the amount of bound mAb (obtained from FCM fluorescence values and autocalibration) as a function of total amount of mAb were analyzed according to Langmuir adsorption expression (see [27]), which enables deriving an affinity constant (*K_a_*) and the number of binding sites per cell, *n*. *K_a_* equal to 2×10^9^ M^−1^ and 6×10^8^ M^−1^ was found for anti-CD3-TCR/CD3 and anti-LFA-1/LFA-1 binding, respectively. Mean number of receptors per cell was equal to (1.2±0.5)×10^5^ per cell and (1.8±0.5)×10^5^ per cell for TCR/CD3 and LFA-1, respectively. Ligand particle surface densities (anti-CD3 and anti-LFA-1) were measured using GAM-Alexa titration. First, particles of increasing mAb surface density were titrated using saturating concentrations of GAM; then, particles saturated with mAbs were titrated using increasing concentrations of GAM. This enabled verification of all mAb coatings with one measurement of GAM-saturated particle fluorescence. Particles grafted with 0.33 mg/ml biotinylated mAb presented a surface density of (1.9±0.3)×10^4^ mAb/µm^2^, i.e.( 4.8±0.5)×10^5^ /particle.

### Cell-particle binding

Cells (5×10^6^/ml) and particles (5×10^7^/ml) were brought into contact at time t = 0 in a 3 ml round-bottom tube at the indicated temperature, usually 37°C, and maintained in suspension using oscillating stirring. Aliquots of 5 µl were taken from the sample at regular time intervals for FCM analysis.

### Ca_i_^2+^ measurements

Flow cytometry Ca_i_^2+^ measurements were performed using the Fluo-3 or Fura-Red calcium probe. Both can be excited by an argon-ion laser at 488 nm. Fluo-3 fluorescence intensity (λ_max_ = 500 nm) increases with increasing calcium concentration [48]. In contrast Fura-Red fluorescence intensity (λ_max_ = 600 nm) decreases with increasing calcium concentrations. Stock solutions of the AM-ester form of the fluorescent Ca^2+^ indicator were prepared in dimethylsulfoxide (DMSO). T cells (Jurkat) were loaded in HBSS with 0.5 µM Fluo-3 or 10 µM Fura-Red for 1 h at 37°C. Typically, 200 µl of T cells (5×10^6^ cells/ml) were loaded. Calibration, enabling linking fluorescence intensity with intracellular concentration, was performed using the calibration buffer kit, exposing calcium probe-loaded cells to buffers which free Ca^2+^ concentration was set between 0 and 39 µM with appropriate EDTA concentrations. In the presence of 10 µM of calcium ionophore A-23187, Ca^2+^ was quickly equilibrated between the outside and the cell cytoplasm and the following equation may be used to determine the ion dissociation constant K_d_,.
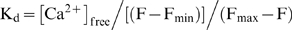
where F_min_ is the fluorescence intensity of the indicator in the absence of calcium (no calcium added; 10 mM EDTA) and F_max_ is that of the indicator saturated with calcium (39 µM Ca^2+^; no EDTA). F is fluorescence measured on the sample in the experiment. In Jurkat cells, we found a K_d_ value equal to 0.9 µM for Fluo-3 and 0.4 µM for Fura-Red. F_min_ and F_max_ were determined for each experiment. Fluorescence intensity of the loaded cells depended both on the incorporated probe and the actual Ca^2+^_i_ concentrations. Within the same cell population, 98% of cells loaded with Fluo-3 had a fluorescence intensity typically ranging from 15 to 350 a.u. (mean FL1 around 175 a.u. corresponding to Ca^2+^ concentration value of 100 nM). The width of the distribution was mainly due to probe concentration variation from one cell to another. Indeed, equilibration of Ca^2+^_i_ with A23187 did not reduce the width of fluorescence distribution, which ranged from 200 to 4500 for mean values close to 1295 a.u.

### Numerical treatment

In a flow cytometry experiment one measures a signal which is the sum of the contributions to the signal given by all the cells activated until time *t*. Each of these cell activated at time *t_0_* gives at time *t a s*ignal *S(t−t_0_)*. Typically the signal measured in flow cytometry is given as normalized by the number of cells activated over the entire observation time *t*. Given the unitary signal *S(t)* and the number of cells *N*, it is therefore possible to compute theoretically the expected signal *C(t)*. The number of particle-bearing cells entering the system in the time interval *dt*, is given by the cell-particle binding kinetics *N(t) = N_0_(1−exp(−kt))* providing *dN(t) = kN_0_exp(−kt)*. Mathematically, calculating C(t) is equivalent to convoluting *S(t)* with cell number kinetics and normalizing the result with respect to *N(t)*:
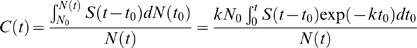
This computation is performed by a home-made program implemented in Matlab. As signal *S(t)* we used the one measured experimentally and for numerical convenience we interpolated it with a cubic spline (a polynomial curve constrained to interpolate all points and formed by piecewise cubic polynomials). Note that in principle we could inversely extract the signal *S(t)* from *C(t)* using a deconvolution algorithm, but this operation gives rather disappointing results due to the experimental fluctuations.

## Supporting Information

Figure S1 Binding time constant dependence of calcium signal shift: (A) Convolution of S(t), the calcium signal instantaneously triggered by soluble anti-CD3 (•) by the time function N(t) = Nmax(1−exp(−kt) reporting the growth of the particle-bearing cell population is shown for increasing values of the time constant k. Corresponding characteristic times (1/k) of 10; 25; 50;100; 250; 1200 s are displayed with increasingly dark grey lines. 1/k = 545 s, corresponding to our experimental situation is shown in red. (B) Signal peak shift is shown as a function of time constant. The main shift actually takes place for time constant values comprised between 0.1 and 3 min-1.
(1.08 MB TIF)

Figure S2 Particle binding and receptor density: Total cell population before particle contact - dot plot FL3 versus FSC shown in (A) - and free cells of a cell-particle sample -dot plot in (B) - were labeled using fluorescent (alexa 488) anti-CD3 (C). FL1 intensity directly reported cell surface density and shows that free cells corresponded to the cell subpopulation of lowest density.
(1.07 MB TIF)

Figure S3 Cell-particle coated with anti-LFA-1 and anti-CD3 antibodies (90∶10 ratio) contact performed using ζCD3-GFP expressing Jurkat cells. Particles were brought into contact with cells and time-lapse images were immediately recorded with a two seconds time-lapse in order to monitor TCR/CD3 surface distribution. Bright field (A) and fluorescence (B) images are shown for times comprised in the first two minutes of the contact, i.e. during the Ca2+i rise time. GFP fluorescence intensity was measured in two equivalent regions located at cell free contour (in black) or at cell-particle interface (in red) for the whole time-lapse stack of images and plotted versus time (C). No significant change in TCR/CD3 distribution was induced at particle contact. images were immediately recorded with a two seconds time-lapse in order to monitor TCR/CD3 surface distribution. Bright field (A) and fluorescence (B) images are shown for times comprised in the first two minutes of the contact, i.e. during the Ca2+i rise time. GFP fluorescence intensity was measured in two equivalent regions located at cell free contour (in black) or at cell-particle interface (in red) for the whole time-lapse stack of images and plotted versus time (C). No significant change in TCR/CD3 distribution was induced at particle contact.
(0.93 MB TIF)

Figure S4 Cell-particle contact area: The contact area was assumed to form a spherical cap with a solid angle of α on the bead. α was estimated on microscope images - we show here a representative example - and contact area was taken equal to 2πrb2(1-cosα/2).
(2.27 MB TIF)
