# Correction: T-Cell Artificial Focal Triggering Tools: Linking Surface Interactions with Cell Response

**DOI:** 10.1371/annotation/d548da9a-692a-4ae9-98ca-92c1ddfa4186

**Published:** 2009-09-16

**Authors:** Benoît Carpentier, Paolo Pierobon, Claire Hivroz, Nelly Henry

Figure S3 is incorrect. Please view the correct Figure S3 here: 
