# Supplementary figures and images for: T-Cell Artificial Focal Triggering Tools: Linking Surface Interactions with Cell Response
Source: PLoS One. 2009 Mar 10;4(3):e4784. doi: 10.1371/journal.pone.0004784 (PMC2653282; doi:10.1371/journal.pone.0004784)

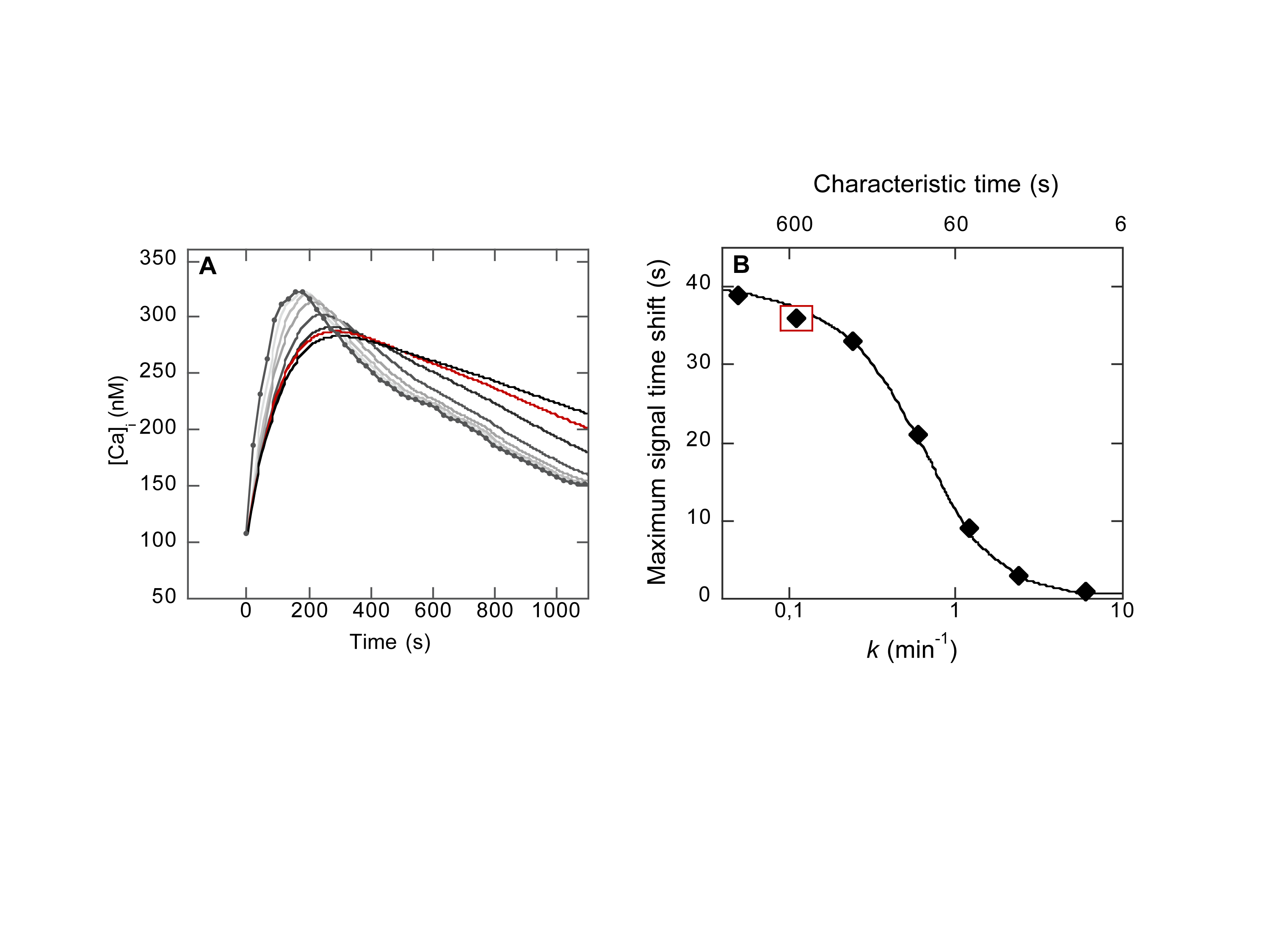

Supplement: Figure S1 — Binding time constant dependence of calcium signal shift: (A) Convolution of S(t), the calcium signal instantaneously triggered by soluble anti-CD3 (•) by the time function N(t) = Nmax(1−exp(−kt) reporting the growth of the particle-bearing cell population is shown for increasing values of the time constant k. Corresponding characteristic times (1/k) of 10; 25; 50;100; 250; 1200 s are displayed with increasingly dark grey lines. 1/k = 545 s, corresponding to our experimental situation is shown in red. (B) Signal peak shift is shown as a function of time constant. The main shift actually takes place for time constant values comprised between 0.1 and 3 min-1. (1.08 MB TIF) [file pone.0004784.s001.tif]

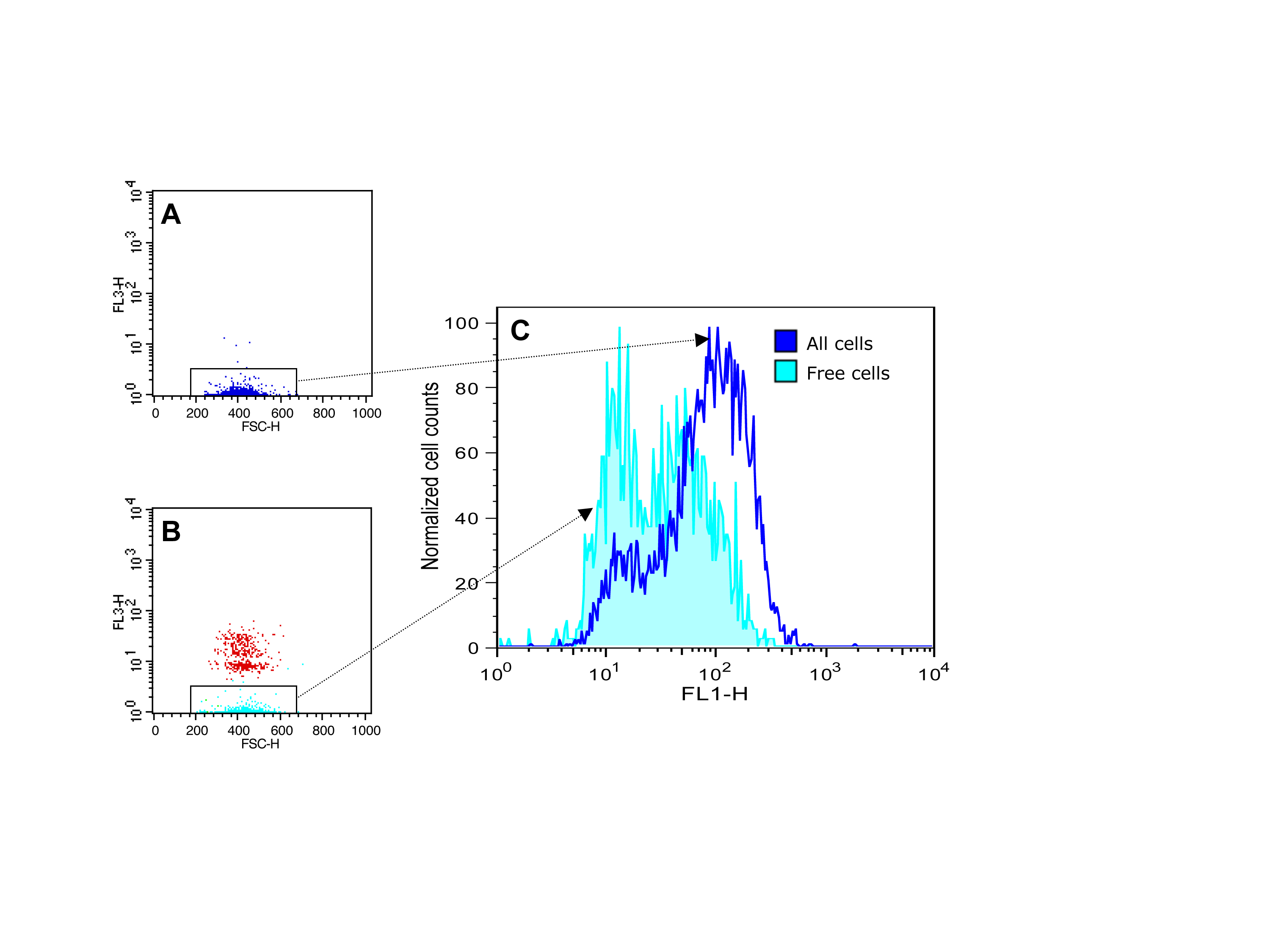

Supplement: Figure S2 — Particle binding and receptor density: Total cell population before particle contact - dot plot FL3 versus FSC shown in (A) - and free cells of a cell-particle sample -dot plot in (B) - were labeled using fluorescent (alexa 488) anti-CD3 (C). FL1 intensity directly reported cell surface density and shows that free cells corresponded to the cell subpopulation of lowest density. (1.07 MB TIF) [file pone.0004784.s002.tif]

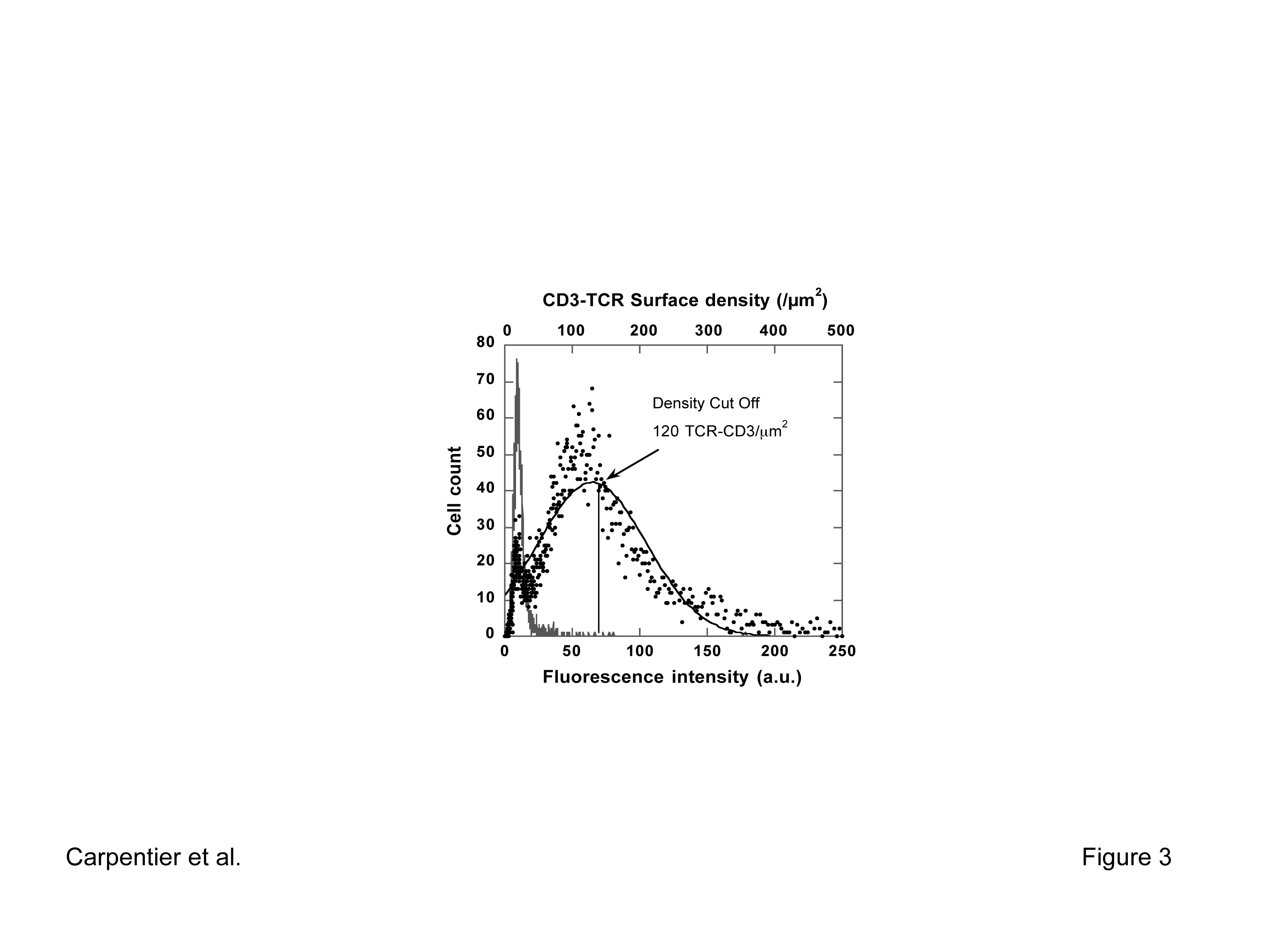

Supplement: Figure S3 — Cell-particle coated with anti-LFA-1 and anti-CD3 antibodies (90∶10 ratio) contact performed using ζCD3-GFP expressing Jurkat cells. Particles were brought into contact with cells and time-lapse images were immediately recorded with a two seconds time-lapse in order to monitor TCR/CD3 surface distribution. Bright field (A) and fluorescence (B) images are shown for times comprised in the first two minutes of the contact, i.e. during the Ca2+i rise time. GFP fluorescence intensity was measured in two equivalent regions located at cell free contour (in black) or at cell-particle interface (in red) for the whole time-lapse stack of images and plotted versus time (C). No significant change in TCR/CD3 distribution was induced at particle contact. images were immediately recorded with a two seconds time-lapse in order to monitor TCR/CD3 surface distribution. Bright field (A) and fluorescence (B) images are shown for times comprised in the first two minutes of the contact, i.e. during the Ca2+i rise time. GFP fluorescence intensity was measured in two equivalent regions located at cell free contour (in black) or at cell-particle interface (in red) for the whole time-lapse stack of images and plotted versus time (C). No significant change in TCR/CD3 distribution was induced at particle contact. (0.93 MB TIF) [file pone.0004784.s003.tif]

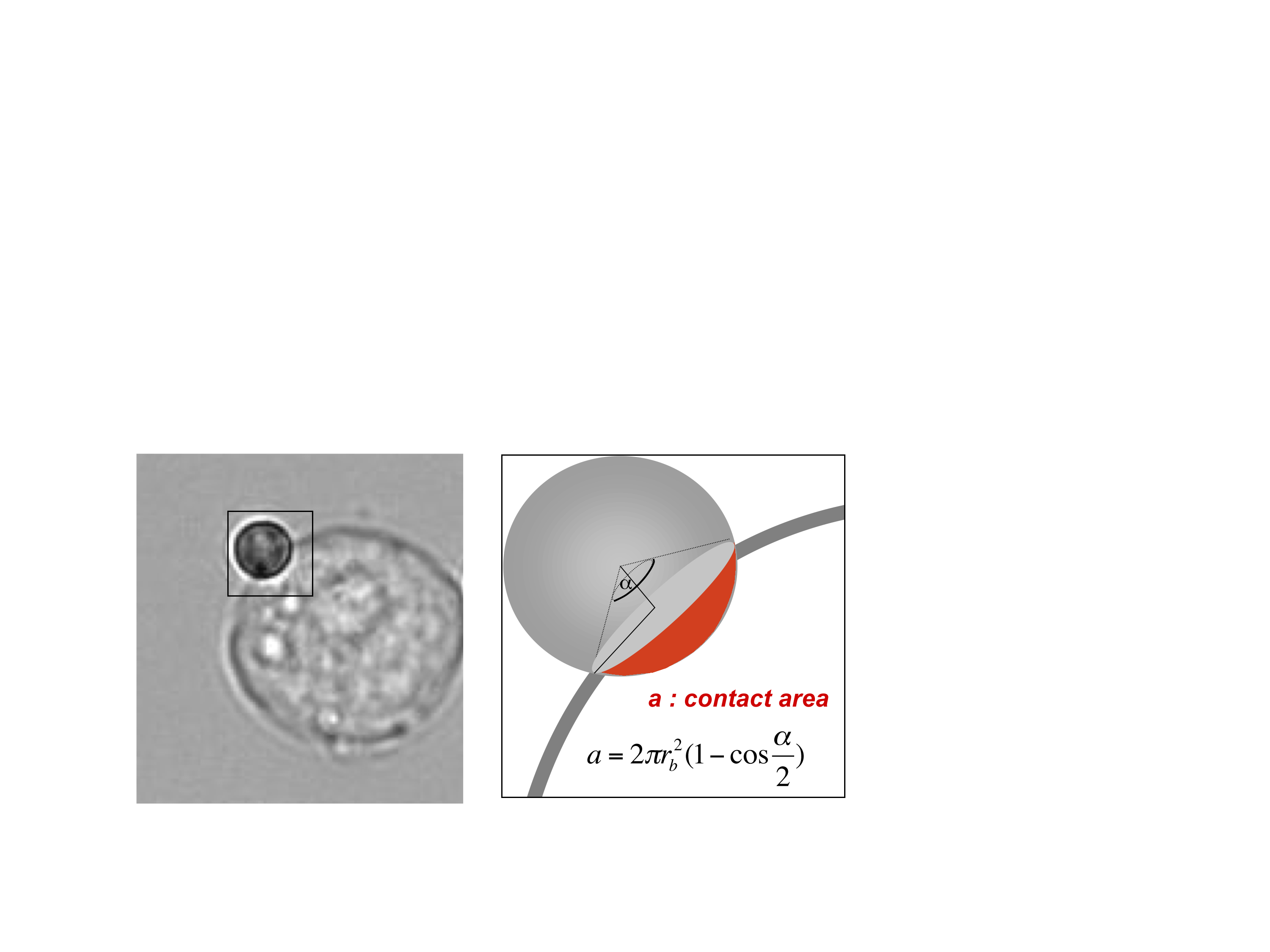

Supplement: Figure S4 — Cell-particle contact area: The contact area was assumed to form a spherical cap with a solid angle of α on the bead. α was estimated on microscope images - we show here a representative example - and contact area was taken equal to 2πrb2(1-cosα/2). (2.27 MB TIF) [file pone.0004784.s004.tif]
