# Supplementary figures and images for: Correction: T-Cell Artificial Focal Triggering Tools: Linking Surface Interactions with Cell Response
Source: PLoS One. 2009 Sep 16;4(9):10.1371/annotation/d548da9a-692a-4ae9-98ca-92c1ddfa4186. doi: 10.1371/annotation/d548da9a-692a-4ae9-98ca-92c1ddfa4186 (PMC2754471; doi:10.1371/annotation/d548da9a-692a-4ae9-98ca-92c1ddfa4186)

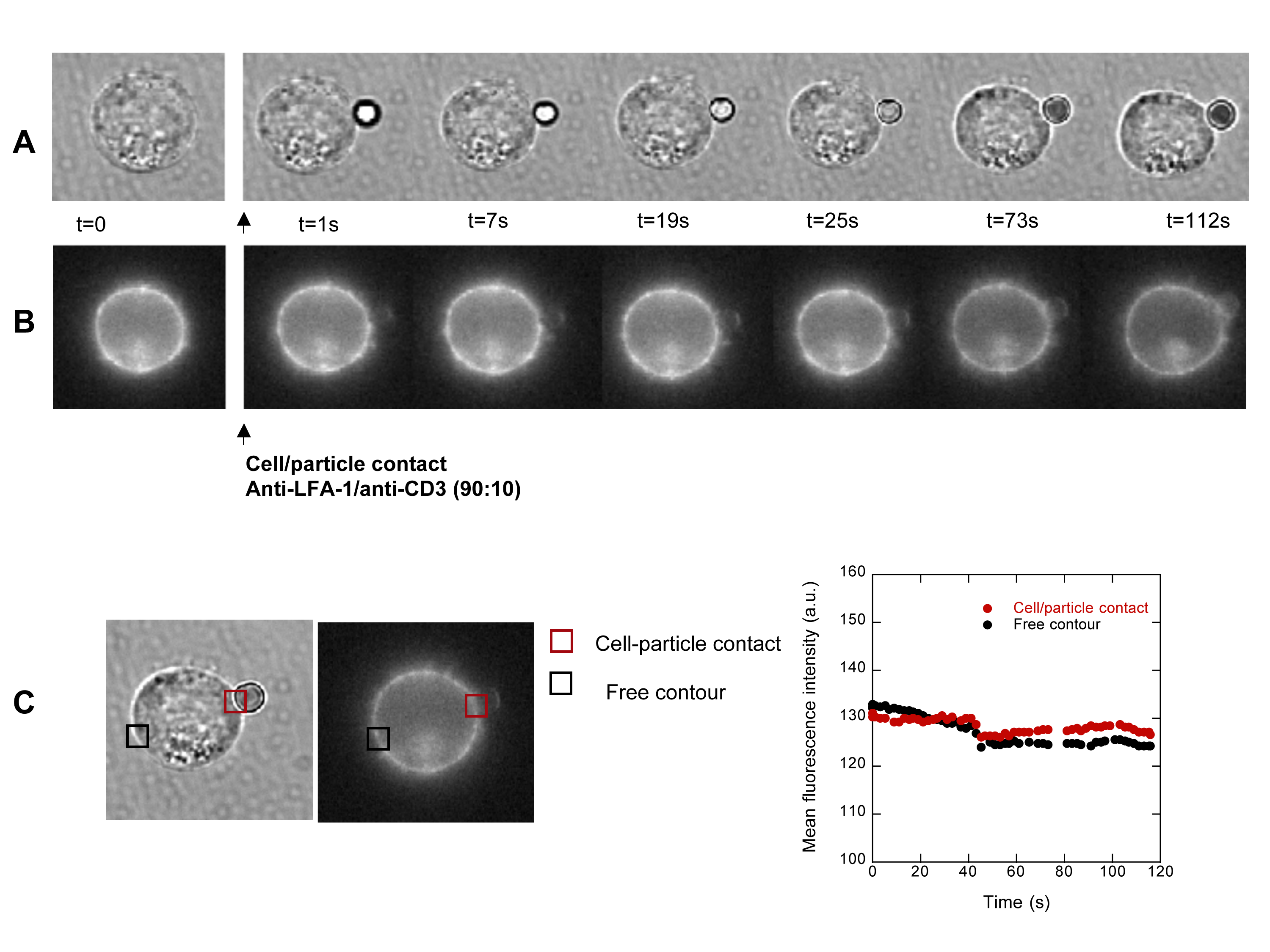

Supplement: Supplementary file 1 [file pone.d548da9a-692a-4ae9-98ca-92c1ddfa4186.s001.tif]
